# Community perspectives on adolescent mental health stigma in American Samoa

**DOI:** 10.1371/journal.pmen.0000080

**Published:** 2024-10-03

**Authors:** Vanessa Blas, Emma J. Mew, Julia Winschel, Leiema Hunt, Si’itia Soliai Lemusu, Sarah R. Lowe, Joshua Naseri, Robert L. M. Toelupe, Nicola L. Hawley, Jueta McCutchan-Tofaeono

**Affiliations:** 1 Department of Chronic Disease Epidemiology, Yale School of Public Health, New Haven, Connecticut, United States of America; 2 Department of Social and Behavioral Sciences, Yale School of Public Health, New Haven, Connecticut, United States of America; 3 Department of Health, American Samoa Government, Pago Pago, American Samoa; 4 Obesity, Lifestyle, And Genetic Adaptations Study Group, Pago Pago, American Samoa; 5 Department of Veterans Affairs, Pago Pago, American Samoa; 6 Empowering Pacific Island Communities (EPIC), Pago Pago, American Samoa; Uganda Martyrs University, UGANDA

## Abstract

Despite reports of mental health concerns among adolescents in American Samoa, little is known about the current mental health burden. Furthermore, previous literature has identified mental illness-related stigma as a significant global barrier to mental health care access and treatment. By gathering various perspectives from adult stakeholders and adolescent participants, this community-partnered qualitative study aimed to describe the perceived stigmatization of mental health in American Samoa. Employing the Pacific-specific *Fa’afaletui* research framework, 28 adult informants of differing professions, ages, and genders participated in semi-structured, in-depth virtual interviews from October 2020 to February 2021. In June 2022, 35 adolescents took part in five online focus groups to validate themes generated from the adult interviews. After duplicate coding of the transcripts, the research team adopted a deductive approach to identify levels of mental health stigmatization before mapping them on to a socio-ecological model. Participants described multiple levels of mental health stigma an adolescent struggling with mental health challenges in American Samoa may encounter. Although there is progress in mobilizing services and educational resources to address various mental health needs, the perceived structural, social, interpersonal, and self-stigma of mental illness may prevent an adolescent in American Samoa from seeking social support and utilizing mental health services. Current and future interventions promoting adolescent mental wellness in American Samoa should focus on addressing the multi-level aspects of mental health stigma.

## Introduction

Maintaining mental health and wellness remains a growing problem for both adolescents and adults worldwide. Since more than half of mental health problems start during childhood and adolescence [1], building mental health literacy and promoting mental health is crucial during this period. In the United States, the prevalence of depression in American adolescents (aged 12–17) increased by nearly 8% from 2009 to 2019 [2].

American Samoa, an unincorporated United States territory slightly larger than the size of Washington D.C., is in the South Pacific with a population of nearly 50,000 people [3]. While previous studies have found that rates of suicide and suicide attempts are alarmingly higher in Pacific Islander youth compared to the Western Pacific region and European regions [4, 5], less is known about the status of mental health among American Samoan adolescents.

Emerging evidence, however, underscores the need to conduct thorough, culturally appropriate research to better understand adolescent mental health in American Samoa. In the 2013 iteration of the Youth Risk Behavior Survey (YRBS) conducted in American Samoa (the most recently available, territory-wide data), 38% of students in grades 9–12 felt so sad or hopeless every day during the past two weeks that they stopped doing their usual activities. In the same survey, 23% of students reported that they contemplated attempting suicide during the past twelve months; higher than the 17% of U.S. high school students who reported they contemplated attempting suicide during the same year [6]. More recently, a cluster of adolescent deaths by suicide shows a mounting public health challenge among American Samoan youth [7].

Although the clinical mental health workforce in American Samoa is limited (two trained psychologists, two psychiatrists, and two licensed clinical social workers [2023]) [8], there are several existing behavioral health services, school-based services (teachers and school counselors), and local non-profit organizations dedicated to supporting youth mental health. Mental illness-related stigma, however, may pose an obstacle to the utilization of these services. The stigmatization of mental illness includes negative perceptions and prejudicial attitudes, labeling, and discriminatory behaviors from the public toward individuals living with a mental disorder [9–11]. Mental illness stigma can manifest at multiple levels of society and be embedded within social institutions and health systems [12]. Individuals facing mental health challenges may experience negative adverse psychological and psychosocial outcomes due to stigma, including loss of self-esteem, poor mental health, and social exclusion [9, 13, 14]. A recent study found that compared to the general U.S. public, Native Hawaiians and other Pacific Islanders (NHPI) reported greater levels of mental illness stigma within their community [15]; it is not clear whether this extends to the American Samoan context.

Embedded in a larger qualitative project exploring the state of adolescent mental health in American Samoa [16], this analysis sought to understand how those in American Samoa perceive the stigmatization of mental health among adolescents, and how multiple components of stigma might negatively affect an adolescent’s relationships with others, conception of self, and accessibility and utilization of mental health services or treatment.

## Methods

Semi-structured in-depth interviews with adult (>18 years) key informants (KIs) living in American Samoa were conducted over Zoom between October 2020 and February 2021. Between June 2022 and May 2022, adolescent (aged 13–18 years) participants were placed in one of five different focus groups on Zoom. Ethical approval was obtained from the Yale University (#2000028354) and the American Samoa Department of Health (#00001249) Institutional Review Boards. Adults key informants provided written informed consent; adolescents provided both their written informed assent and written parent/guardian consent for their participation.

The semi-structured interviews and focus group discussions used open, non-directive questions that focused on defining mental health and mental illness, broad topics around common mental health problems, barriers and facilitators to mental health care, and potential interventions to improve the current mental health prevention and treatment infrastructure (interview guides in Tables 1 and 2). Further methodological information has been reported previously [16].

**Table 1:** Perspectives of 28 adult key informants according to the *Fa’afaletui* research framework.

KI #	Perspective
1	Top of the mountain
2	Person in the canoe fishing
3	Top of the tree
4	Top of the mountain
5	Person in the canoe fishing
6	Top of the mountain
7	Top of the mountain
8	Top of the mountain (x2 participants)
9	Top of the tree
10	Top of the mountain
11	Top of the mountain
12	Top of the tree
13	Top of the mountain
14	Top of the tree
15	Top of the mountain
16	Top of the mountain
17	Top of the mountain
18	Person in the canoe fishing
19	Top of the tree
20	Top of the tree
21	Top of the mountain
22	Person in the canoe fishing
23	Top of the mountain
24	Top of the tree
25	Top of the tree
26	Top of the mountain
27	Top of the tree

*Note*. Each perspective represents differing professions and backgrounds—‘the top of the mountain’ (e.g. policy makers, non-governmental organizations, and mental health providers), from ‘the top of the tree’ (i.e. adults who work directly with adolescents, for example teachers, extracurricular program leaders, etc.), and ‘the person in the canoe fishing’; those most impacted, i.e. young adults).

**Table 2:** Adult semi-structured interview guide topics and sub-questions.

	Topic	Example Sub-Questions
1	Opening Question	Can you tell me a little bit about your thoughts on the state of mental health among adolescents in American Samoa?
2	Status of Mental Health	What are the most common mental health problems among adolescents in American Samoa, if any at all?
3	Signs and Symptoms of Mental Distress	What are the signs that an adolescent in American Samoa is struggling with mental health that someone else might notice?
4	Predictors of Mental Health and Illness	What do you think is causing adolescent mental health problems in American Samoa? What would make an adolescent have good mental health?
5	Initial Point(s) of Contact and Care Pathways	What paths do adolescents in American Samoa struggling with mental health problems follow in the course of their search for help?
6	Coping Mechanisms for Mental Distress	Would you know of any coping mechanisms that adolescents struggling with mental health problems in American Samoa use to feel better?
7	Existing Infrastructure	What services are available to help adolescents living with mental health problems on Island?
8	Barriers and Facilitators to Care	What might prevent an adolescent from using these resources? What might make it easier for an adolescent to use these resources?
9	Potential Interventions	If you could create a program or a change of some kind, what do you think would most improve adolescent mental health in American Samoa, if any at all?
10	Session Closing	Are there things you think we didn’t cover today that are important about adolescent mental health that we should know about?

### Key informant interviews (Adult stakeholders)

Adult key informants were initially nominated by partners at the American Samoa Department of Health and Division of Behavioral Health Services; additional participants were identified using snowball sampling. Speaking English was a requirement for participation, although likely contributed very little bias since >80% of the general population and almost all those in professional roles speak English fluently [3]. While identifying as Samoan was not an inclusion criterion for adult participants, the research team recruited participants of Samoan ethnicity when possible.

To sample for diversity in community perspectives, the *Fa’afaletui* methodological framework, developed for research in Samoan communities [17, 18] was used. This framework prioritizes the recruitment and weaving together of varying views on adolescent mental health, from the broader outlook ‘the top of the mountain’ (e.g., mental health providers, policymakers, and those working in non-governmental organizations), the mid-distance lens ‘from the top of the tree’ offered by individuals who work directly with adolescents like teachers, and the finer observations ‘from the person in the canoe fishing’ that young adults and adolescents could provide [17, 18]. The MacDonald et al. [19] framework on pathways to mental health services for young people was also followed to maximize diversity when recruiting participants and to ensure that various professions with different degrees of involvement along the mental health services pathways–from mental health professionals to school staff and religious leaders–were included. Aligning the MacDonald et al. framework with the *Fa’afaletui* framework resulted in a sampling frame that aimed for diversity by age, gender, region, residence, and education level.

Fifty-six individuals and four organizations were invited to participate; 36 KIs (64%) expressed interest in participating. Of these, eight KIs were unable to participate because of scheduling problems or due to the sample reaching saturation. A total of 28 KIs therefore comprised the final adult sample.

### Adolescent focus groups

Five focus groups were conducted between May 2022 and June 2022 with English-speaking school-age adolescents. The primary aim of these discussions was to validate the results gathered from the adult interviews. To participate in the focus groups, adolescents were required to identify as ethnically Samoan and have lived in American Samoa for at least one year. Participants were primarily recruited using a Facebook advertisement, supplemented with snowball sampling using email, texts, and Facebook messenger leveraging our collaborators’ and participant’s social networks.

Thirty-five adolescent participants were in the final sample. We convened one group of all boys, two with all girls, and two mixed gender groups. Each focus group session was led by two moderators from American Samoa who are Samoan, speak Samoan, and have expertise in Samoan culture. Although interview prompts were mostly read in English, the moderators used English and Samoan languages based on participant preference.

### Analysis

Interviews and focus groups were transcribed verbatim with the assistance of Temi software [20]. Deductive thematic analysis was primarily used to identify explicit meanings of the data. Most frameworks on health-related stigma focus on the individual experiencing stigma and/or those who stigmatize an individual [21, 22], but more researchers in high-income countries (HIC) and low- and middle-income countries (LMIC) are now addressing the individual, social, and structural pathways in which stigma manifests through a socio-ecological framework [21–24]. Therefore, a socio-ecological model was used to guide the assessment of mental health stigma into different forms or manifestations: structural stigma, public (or social) stigma, interpersonal stigma, and internalized (or self) stigma. Employing this framework allowed for the visualization of the effects of stigma across multiple domains and different populations, particularly adolescents. Evaluating the different levels of mental health stigma may help organizations, community leaders, and advocates develop interventions that target stigma at certain or multiple levels. Illustrative quotes are included to demonstrate the depth and breadth corresponding to each level of mental health stigma. To identify the characteristics of the 28 adults and 35 adolescents who participated in our interviews and focus groups, respectively, the following labeling system was developed. Adults are referred to as “A” and adolescents–or youth–are referred to as “Y”. For the adult participants, an uppercase superscript was included to identify whether they represent perspective from the top of the mountain (“M”), top of the tree (“T”), or person in the canoe fishing (“C”); for adolescents, a lower-case superscript was included to identify their gender as male (“m”) or female (“f”).

## Results

Among the 28 adult KIs, 13 were mental healthcare providers–specifically, four licensed mental health professionals and nine lay counselors–while the remaining 15 were community members [16]. Each KI brought diversity in perspectives from ‘the top of the mountain’ (n = 15), ‘the top of the tree’ (n = 9), and ‘the person in the canoe fishing’ (n = 4), with the four KIs in the ‘canoe’ being young adults. The 35 adolescent focus group participants (with an age range of 13–18 years, the average age being 15.8 years) provided additional perspectives from the ‘person in the canoe fishing’. All adolescent and young adult participants, and most of the adult stakeholders, were of Samoan ethnicity. No other demographic characteristics are described, because of the possibility of identification in a small, island community.

**Fig 1:**
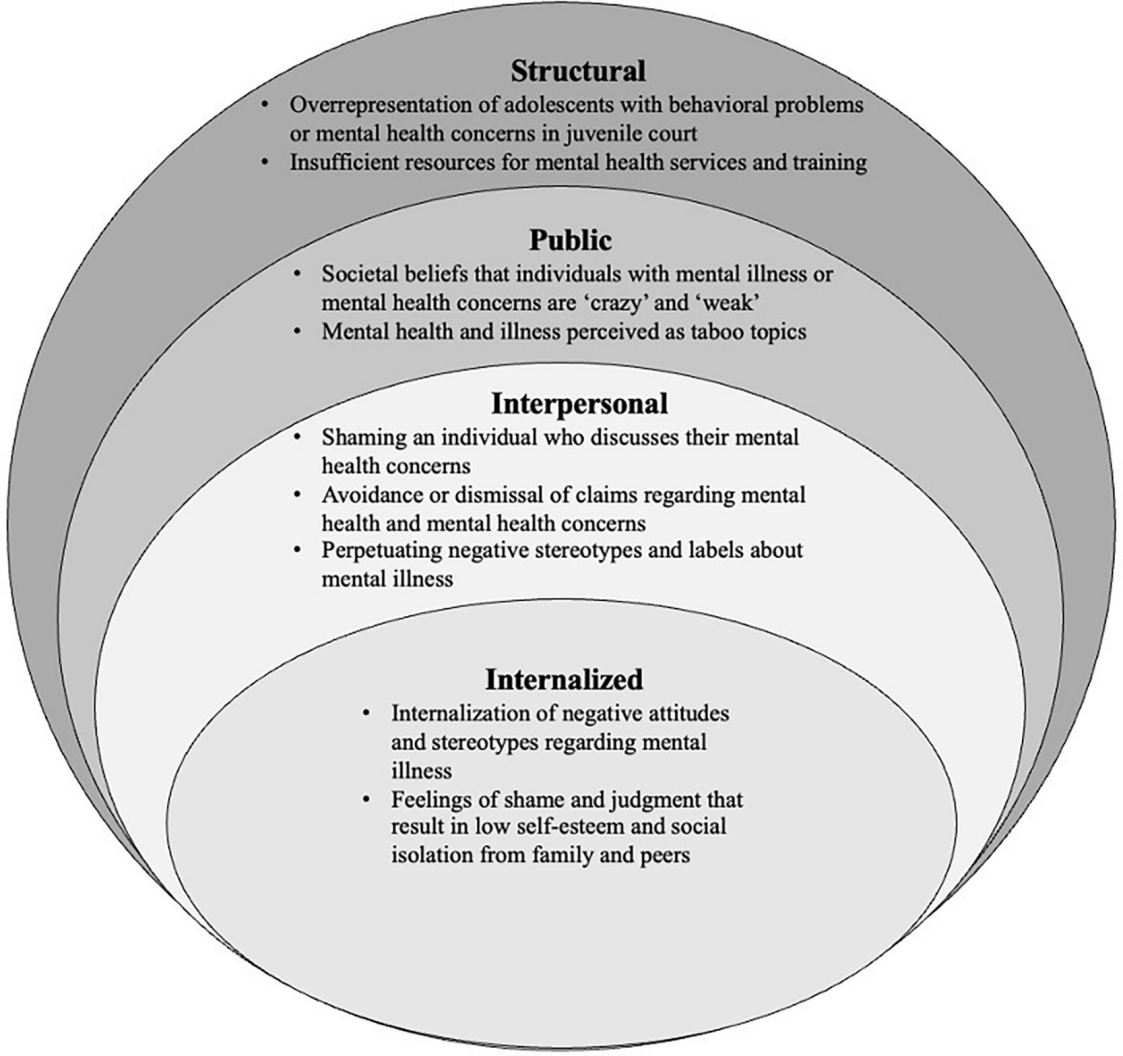
Socio-ecological model of mental health stigmatization for adolescents in American Samoa. Based on the qualitative interviews, perceived manifestations of mental health stigma for adolescents in American Samoa at the individual, interpersonal, public, public, and structural levels were mapped on a socio-ecological model.

Adolescents largely validated the themes identified in adult interviews. Importantly, both adults and adolescents shared a common understanding of mental health and illness that was aligned with current medical standards and interpretations. Adult KIs and adolescent focus group participants explicitly and implicitly described multiple forms of mental health stigma among adolescents in American Samoa. Based on their accounts, each level of stigma is influenced by and interacts with each other, potentially resulting in negative psychosocial outcomes for a stigmatized adolescent and/or hindering an adolescent from seeking social support and utilizing any mental health services (**Fig 1**).

### Structural stigma: “Human capital is really limited” (A-ID#10^M^)

Structural stigma refers to institutional policies and societal-level conditions that result in unfair treatment of the individual experiencing stigma. Multiple adult KIs cited a lack of investment by policymakers in mental health infrastructure, reflected in insufficient funding and resource allocation toward mental health services, especially in the school system: “*Unfortunately*, *it has been years since we have had adequate training to actually empower [school] counselors*…*what has happened over time is that we have school counselors who are school counselors*, *on paper*, *but they do not have the skills*, *nor do they possess the abilities from the Department of Education to actually deal with some of the mental health issues*” (A-ID#07^M^).

While one adolescent participant mentioned a positive experience with their school counselor (Y-ID#18^m^), many other adolescent participants expressed their disappointment with high school students receiving a lack of mental health support *“because of an inactive counselor”* (Y-ID#20^m^) or because counselors are *“not able to see that our kids are struggling”* (Y-ID#22^f^).

*“My counselor has had a lot of students come into her office for acting out*, *but she doesn’t see that if she could actually talk to these kids*, *she would actually know the reason and purpose as to why these kids act out*, *why they misbehave in class instead of labeling them as the bad kids or the naughty kids”* (Y-ID#20^m^).

Adult KIs also commented on the limited number of mental health professionals in American Samoa, believing the current number is, “*not enough to do assessments for all the kids…I think that there’s a lot [mental health conditions] that’s out there that’s just not been identified or diagnosed”* (A-ID#04^M^).

Many KIs often mentioned the lack of a clear referral system for adults to follow if they were concerned about an adolescent struggling with a mental health challenge: “*The way the system works now*, *it’s only those who…have severe behavioral problems”* (A-ID#01^M^).

Adult KIs also explained the roles of legal authorities and the court system when attempting to find resources for an adolescent who may be struggling with behavioral and/or mental health issues: *“…Instead of possibly going into the hospital*, *to look for help*, *it’s more of arresting the person and seeing them stay in prison rather than seeking a…mental health professional to see if maybe possibly they have a mental health condition”* (A-ID#21^M^).

One KI described a negative experience with police involvement after the KI had expressed concern about a student’s signs of self-harm to the school administrators:

*“I’m just going to take [the student] with me…went to the principal’s office*. *He called the police…[they] came in and then started questioning her…I’m like*, *‘Oh my goodness*, *she’s the victim here*? *Why are you treating her like she is the abuser*?*’…Where are the social workers*? *Is there a psychiatrist*?*”* (A-ID#11^M^). Another KI commented on the need to strengthen school mental health screenings because, “*[adolescents] who…have had a long history of school disciplinary problems…are generally not picked up and…reach mental health services until they are involved in the legal system”* (A-ID#01^M^).

The lack of sufficient funding for mental health care in American Samoa also limits the availability of educational resources on mental health. This form of structural stigma drives the stigmatization of mental health and illness at the public, interpersonal, and individual levels, ultimately shaping the way mental health is perceived among the community and communicated between adolescents and their families. Both adult KIs and adolescent participants alike frequently cited a lack of mental health literacy and education among the community, schools, and families in areas such as:

1. how to healthily express one’s emotions: “*It’s because [adolescents are] depressed and they don’t really know how…to express themselves or…talk to someone”* (A-ID#23^M^).
2. knowing whom to go to for mental health support: *“*…*when someone falls down at school and breaks their leg*, *who do you call*?*’*, *and you’re like*, *‘the ambulance’*, *they know*. *‘But if you have something that’s bothering you emotionally*, *who do you call*?*’ Everybody just kind of looks around*, *and ‘I don’t know’”* (A-ID#13^M^).
3. maintaining mental wellness: *“We don’t see that we need to have a solution…to help ourselves*…*And we’re just blinded to the fact that we are mentally unhealthy”* (Y-ID#16^f^).

Several KIs listed recent efforts to combat mental health stigma by increasing mental health awareness among the American Samoan population and encouraging community input. For instance, these two cases show efforts from the government and church community, respectively: *“*…*a Task Force for Suicide Prevention just started…headed by the Department of Health*, *with mental health services…presentations in school and [going] to groups in villages and talk to parents and children*, *so…the adolescents would get information from that public awareness*.*”* (A-ID#16^M^; note, minor edits were made to remove potentially identifying information)

*“There’s just been this huge outpouring of community involvement…being spearheaded by this particular Christian pastor*. *And we’ve seen positive feedback from the kids who are telling their school principals and their school teachers*, *that there needs to be more…opportunities for them to speak and to feel heard and safe spaces…”* (A-ID#07^M^).

Additionally, a young adult KI listed the church community as a positive source of social support to an adolescent who may be struggling with their mental health: “*I think a lot of kids turn to the church because they feel like the church won’t judge them”* (A-ID#02^C^).

### Public stigma: “We just don’t have enough discussions about it” (A-ID#15^M^)

Defined as the cultural norms and negative attitudes the public may hold toward individuals living with mental illness, adult and adolescent participants described the effects of public mental illness stigma in American Samoa, in terms of mental health not being “*openly talk[ed] about*. *We don’t talk about depression and anxiety and stress*. *It’s…wrapped up tightly*, *put in a box and left for later*” (A-ID#13^M^).

Adult KIs outlined two potential ways the broader American Samoan community may respond to topics on mental health and illness: 1) being “*taught that [mental health problems don’t] exist”* (A-ID#14^T^) or 2) having it *“swept under the rug”* (A-ID#03^T^).

There was consensus among adult KIs and adolescent participants that mental health in American Samoa is seen as “*such a taboo topic*” (A-ID#02^C^), despite adolescents agreeing that “*mental health should be prioritized”* (Y-ID#14^f^) and *“most teens are unhealthy mentally”* (Y-ID#18^m^). Multiple KIs believed this negative perception of mental health meant that “*there’s not really a culture of…open dialogue*, *especially when it comes to emotional issues*” (A-ID#14^T^). Adult informants also stated that the topic of mental health was seen as “*Palagi [foreign]*, *not Samoan*” (A-ID#10^M^, A-ID#14^T^) and “*a white person thing*” (A-ID#05^C^).

Some mental health topics the public might find *“disrespectful to talk about”* (A-ID#15^M^) include depression and suicide, though some community leaders reported trying to increase opportunities for education: “*We’re trying to…create a space to talk about suicide”* (A-ID#12^T^). When mental health is discussed in the community, several adult KIs stated it was often done in a negative light: “*in Samoa*, *we have this stigma… that mental health*, *anything pertaining to mental health means that someone’s crazy*” (A-ID#21^M^). Anyone who might show signs of struggling or living with *“a mental health problem*, *is just labeled as crazy*” (A-ID#22^C^).

Most KIs noted how the stigmatizing nature of mental illness in the community can be linked with individuals being “*…not informed…we don’t know about mental health from a very young age*” (A-ID#12^T^). An adolescent stated this may prevent students from knowing “*where to seek help from*” (Y-ID#25^m^). Many KIs commented the broader community’s “*lack of education on the island about mental health issues*” (A-ID#07^M^) also means the population is “*missing the plain language…And we’re asking people to try to measure their feelings…but not giving them all the tools to be able to do that*” (A-ID#11^M^).

Increasing opportunities for mental health education would require policymakers and community leaders in American Samoa to address structural stigma and increase cross-sectoral collaboration, funding, and resource allocation for mental health programs, a need that one adult KI stressed: “*I would like to see some kind of organized*, *consolidated collaboration by churches*, *NGOs*, *and the government…because there’s just a lot of fragmentation right now*…” (A-ID#07^M^).

Additionally, adult KIs and adolescents described a “social pressure” that hinders individuals from disclosing how they may be feeling, especially among men and boys, as one young adult mentioned how “*it’s not masculine to talk about your feelings*” (A-ID#05^C^). Another young adult KI stated that many boys in the community are “*taught to be strong for their families and we need to suck it up and move on*” (A-ID#18^C^), a sentiment that was echoed by multiple male and female adolescent participants:

*“I feel like boys*, *we’re misunderstood sometimes…we’re supposed to be tough…but sometimes we need a break and sometimes we have our moments where we can’t hold the load*.*”* (Y-ID#6^m^) *“But the boys…if you’re too emotional*, *then you’ll be judged by a lot of boys*, *especially if they’re your friends*. *The boys feel like they can’t be emotional”* (Y-ID#11^f^).

Some adult KIs explained how gender roles shape perceptions of mental health and emotional well-being among adolescent boys: “…*the male Samoan male archetype is tough exterior…whatever comes along*, *just grin and bear it… put it in a box*…*deal with it some other time*” (A-ID#13^M^). The majority of KIs perceived that gender-related differences in emotional expression are influenced by cultural norms, and these differences may affect an individual’s ability to find social support when facing a significant stressor or when needing to address a mental health-related need: *“Men aren’t supposed to show feelings*…*more so here in a Pacific culture where everything is [a] patriarchal hierarchy*. *And so young men don’t feel comfortable sharing their feelings with other men or other peers…they just seem to bottle up a lot of that aggression or a lot of those negative feelings that they have until they burst…I feel like for young women*, *they’re able to reach out*, *they have the capacity to reach out to their peers*. *And for young men*, *there’s always this*, *stigma*, *oh ‘I’m a guy*, *I’m a man I’m not supposed to share show or share any feelings*.*’ And so that becomes an obstacle for them to seek the kind of help that they need”* (A-ID#07^M^).

Multiple adult KIs also discussed the role of religion in the community’s perception of mental health: “*There’s a lot of people on [the] island who think that if you say you’re depressed or that you’re going through depression*, *there’s a lot of people who think that that’s all like the devil’s work”* (A-ID#10^M^). While several KIs cited the church community as an important source of support for an adolescent struggling with their mental health, others noted “*the role that the church plays …could be another one of those areas where discussion of themes such as mental health is discouraged”* (A-ID#13^M^). An adult KI further expressed concern over *“this minimizing attitude towards [mental health] as if*, *‘Okay*, *let’s not talk about it*, *let’s pray about it’”* (A-ID#25^T^). A young adult KI held similar thoughts: “*Everybody tells you to turn to God…we’re already doing enough to make them feel like they should pray instead of talking to somebody*” (A-ID#05^C^).

Most KIs believed that the negative public perception of mental illness may affect an adolescent’s interactions with friends and families–described as interpersonal stigma–while also preventing them from seeking help for any issues related to mental health: “*[Adolescents] don’t feel safer at home and now there’s nobody else to talk to at the school level*. *They don’t feel comfortable talking to adults in their village*. *So*, *the question becomes*, *who are they talking to*?” (A-ID#07^M^).

KIs also observed that some of the public’s most recent response to the cluster of suicides in the community may further stigmatize mental illness: *“The public was responding to the recent suicides here on [the] island and there was a lot of victim shaming and victim blaming going on social media*. *So [adolescents who had attempted suicide] were feeling defeated or they were feeling like they were back in that place because the stigmas are still alive and well here”* (A-ID#03^T^). Furthermore, an adolescent was concerned that certain classmates who had posted signs of suicidal ideation on social media were not treated or heard seriously because “*sometimes people would think it’s for attention*” (Y-ID#29^f^).

The perceived stigma around mental health is why adult and adolescent participants stressed the need to increase mental health literacy among the adult and adolescent population in American Samoa: “*Yes*, *everyone is aware of suicide and mental health but they’re not understanding the perspective or view from a person who has thoughts of committing suicide…*. *I feel that people should be educated on what depression is and how we can help with it*, *instead of saying ‘I wish I can help’ or ‘You’ll get through this’*. *Our youth just needs someone to listen to*, *and when I mean to listen*, *I mean actually listening”* (Y-ID#21^f^).

### Interpersonal stigma: “People see mental health as a weakness” (A-ID#25^T^)

Interpersonal stigma refers to interactions that occur between a non-stigmatized individual and a stigmatized person. Adult KIs and adolescents described a myriad of outcomes that may occur if an adolescent struggling with their mental health confides in their parents on how they are feeling. The first three were most frequently mentioned, but references to physical punishment were also common:

1. Shaming: “*It’s frowned upon…If the parents perceive [their children]*, *they’re not in their place*. *They’ll say that*, *you know*, *like* fiapoko *[smart aleck*, *know it all] …*” (A-ID#15^M^)
2. Parental dismission of claims: “*…when they do turn to their parents*, *it’s not a very pretty outcome…they kind of just push it aside…where it’s like*, *‘If you’re an abled body person*, *then why are you depressed*?*’*” (A-ID#05^C^)

*“Whenever I try sharing with my family, they always say mind over matter and just get through it.”* (Y-ID#11^f^)

3) The usage of negative stereotypes associated with mental illness: *“…My students had been sharing that they know a lot of youth who don’t speak up because they’re either shamed for it …their parents would tell them they’re just being lazy or cowardice”* (A-ID#03^T^).
4) Physical punishment: *“We*, *all Samoans*, *we spank our kids*, *so they would*, *be obedient*. *They’ll learn to listen and obey…*. *It [spanking] was like our way of discipline here on Island”* (A-ID#18^C^).

Participants listed the pressure of fulfilling familial expectations as reasons why an adolescent may not feel comfortable discussing their emotions and mental well-being with parents: *“Our culture is brought up in such a way that you’re supposed to be mentally fit and strong to overcome anything*. *I’ve also come across some parents who will not acknowledge the fact that their child has mental health problems*, *that they’re actually going through depression and suicidal tendencies*. *They won’t see it*, *or they won’t acknowledge it*…*”* (A-ID#07^M^).

*“..the fact that some families pressure their kids to achieve more even if that means putting their mental health at risk*” (Y-ID#13^f^).

Several KIs said families may also be influenced by the public stigma around mental health to the point that they *“would rather brush the behavior [of the adolescent] under or hide it under the rug rather than*, *bring it up to light and asking for help from professionals”* (A-ID#21^M^).

KIs and adolescents stated the negative interactions between parents or peers and a stigmatized adolescent may make the adolescent less likely to ask for help in dealing with a mental health issue: *“Kids are taught to toughen it out…when you’re struggling and especially if you’re crying about something*, *there’s not really the process*, *‘Let me help you organize your thoughts and help you feel and calm you down’*… *A lot of it…it’s invalidating*, *‘Why are you crying*? *That’s not even a big deal*. *It’s not anything you should be crying about’*.*”* (A-ID#14^T^).

“*I think [adolescents] should open up, but then again, most of the boys, every time we open up to our parents and some of the adults, most of them will judge us, calling us weak and calling us girls, cause most of them think that boys should always be tough in life”* (Y-ID#2^m^).

Adult stakeholders, including young adult KIs, have also described instances of adolescents being shunned or bullied on social media after showing signs of suicide ideation or self-harm: “*Someone…already feeling weak for having suicidal thoughts and then going on Facebook and seeing people write things about other youth who have committed suicide*, *‘They took the easy way out’ or*, *‘They were cowards’*…*It discouraged [adolescents] to even speak out*” (A-ID#03^T^).

*“Because [self-harm] is a taboo thing here and a lot of kids are made fun of for self-harm, instead of comforted. I know there are kids here who post self-harm and a lot of other kids… screenshot that and share it amongst friends…calling them weak”* (A-ID#02^C^).

Some adolescent participants also noted similar negative experiences with school counselors: “*My counselor likes to yell at me every time when I try to come to her for help*!*”* (Y-ID#17^m^). An adult KI also remarked on a similar moment: *“And one of our counselors is saying*, *‘Why are you crying like a baby*?*’… just ‘Suck it up’… They’re not very sensitive to what the adolescent is going through”* (A-ID#13^M^).

These potential instances of interpersonal stigma an individual with a mental health concern may experience can influence whom they choose to confide in and whether they seek a mental health professional. For example, both adult KIs and adolescent participants believed that adolescents’ concern over confidentiality may prevent them from seeking help or utilizing local mental health services: *“Nowhere have I had more questions about confidentiality than here in Samoa*. *And it makes sense because*, *you know*, *it’s a small place…so that question of where does my information or material*, *where does it go*? *Who do you know*, *who are you going to tell*?*”* (A-ID#10^M^).

Interpersonal stigma may also negatively influence the self-image and self-esteem of a stigmatized individual, potentially resulting in the manifestation of self-stigma.

### Self-stigma: “We don’t actively seek help, even though we might be suffering mentally” (Y-ID#13^f^)

Internalized stigma, or self-stigma, occurs when an individual living with a mental illness holds negative attitudes about their self and shames themselves for their mental health condition or struggles. Adults KIs and adolescent participants frequently described how adolescents may be struggling with addressing their emotional needs while juggling the social pressures they feel from their families, peers, and the broader community, at the expense of their mental health:

“…*expectations that may be had by family members…not wanting to disappoint their family members, feeling a huge sense of responsibility…but this feeling that they’re taking on a lot…they can’t really express themselves to others…[and] needing to kind of create this façade*” (A-ID#10^M^).

*“…making my parents proud is a lot of pressure*. *I do love my parents*, *but the consequences are too much for me*. *I have to meet their every request*…*It is a battle to myself that I have to meet everything my parents want from me just to make them proud but for me*, *it is not particularly what I want for myself*. *So it comes with being depressed and stressed trying to do something that I do not want to do yet it is what I feel like I have to do just to make my parents proud”* (Y-ID#17^m^).

Aside from adolescents potentially hiding their mental health struggles to preserve a positive image for themselves and their families, one adolescent participant also explained that the social pressure by the community to be perfect may be associated with poor mental health among adolescents: *“I don’t really think our youth is mentally healthy mostly because of the impact from social media and also all the pressure that we get from our parents*, *of them wanting us to be perfect*, *wanting us to set an image that sometimes we think we can’t fulfill or that we can’t reach*.*”* (Y-ID#15^f^) Two other adolescent participants agreed with this sentiment and the effect of internalizing negative attitudes on an adolescent’s mental health: *“Pride…always stop me from asking people for help even though I know I desperately need it…that I may look weak or stupid*. *Pride in my family that it might be a disgrace that I cannot stand on my own*. *Pride as a guy that people always expect us to be the stronger person”* (Y-ID#17^m^).

“*You gotta always watch what you’re doing, make sure you don’t take the wrong path…And all you’re gonna think is…’Why am I even doing this when all I’m gonna get back is scolding?’ We can all feel that one mistake…all you can feel is, ‘I’m not even good enough for this family, that’s why they keep lecturing me.’ And that’s where this bad mental health comes in”* (Y-ID#33^f^).

Adolescent participants also commented on the fear of judgment if they tell family members how they are feeling, resulting in a ‘bottling up’ of emotions that negatively affects their mental health: “*In some cases [adolescents] really see it as the only option to turn to suicide…because opening up would mean admitting one’s vulnerability and*, *possibly being subjected to criticism due to their quote unquote ‘lack of strength’*.” (Y-ID#13^f^) Additionally, several adolescent KIs believed that the current actions adolescents take to keep emotions to themselves may prevent the utilization of current mental health services: *“The mindset many of our youth have that they can just keep all their emotions to themselves instead of expressing them…I’m guessing that’s why most of the youth don’t want to dial the [hotline crisis] numbers…because even at our school…they give out the numbers…they give out the pamphlets…but it’s just that some of the students just think…they don’t need people to listen”* (Y-ID#14^f^).

## Discussion

To our knowledge, this study is the first to document the multiple forms of stigma associated with mental illness in American Samoa and its potential effects on adolescents who may be struggling with their mental well-being. These findings provide evidence that any programs or interventions focused on adolescent mental health concerns in American Samoa must also address the stigmatization of mental illness.

Research has shown that high mental health stigma has been associated with low mental health literacy [25–27], bolstering the need to increase mental health campaigning and the inclusion of activities and resources on health literacy in future mental health interventions. Increasing mental health literacy and awareness may be particularly useful in addressing the mental health challenges and concerns among Pacific Islanders, specifically American Samoan adolescents. A qualitative community-based study found that a low level of mental health awareness contributed to mental health stigma among Samoan Americans in Southern California, posing a significant barrier to formal help-seeking [28]. Focus group sessions among Samoan mental health providers and service users in New Zealand similarly found that mental health stigma may prevent Samoan youth from utilizing formal mental health services, with participants highlighting the need to identify de-stigmatization strategies that educate families, churches, and communities about mental health through appropriate Pacific specific language [29]. A recent qualitative study also applied the *Fa’afaletui* framework to investigate Samoan families’ experience with mental health services in New Zealand and found that mental illness stigma posed a significant issue for engagement with mental health services. The authors recommended building capacity for holistic Pacific models of care [30]. Although most research on mental health literacy has been conducted in HIC, literature on the subject is increasing in LMIC due to active collaborations between HIC and LMIC [31].

During the interviews and focus groups, adult and adolescent participants underscored the need to increase mental health literacy to promote mental wellness and behavioral services accessibility in their communities, a recommendation several organizations in Pacific Island communities have also mentioned. In 2003, the Samoa Nurses Association, New Zealand International Aid and Development (NZAID), and the Foundation of the Peoples of the South Pacific International collaborated on the Youth and Mental Health (YMH) Project, which sought to increase mental health education and awareness through advocacy programs and focus group discussions among Pacific youth in Samoa, Papua New Guinea, Vanuatu, Solomon Islands, Kiribati, Tonga, Fiji, and Tuvalu. Youth participants for the YMH project in Independent Samoa acknowledged how social networks provided support and shaped their self-perception [32]. YMH project researchers recommended that interventions focused on mental health promotion must consider how Samoan cultural beliefs and values of religion and family may provide both avenues of resilience among youth as well as additional stressors for individuals whose actions and self-perception are strongly influenced by their peers and family members [32]. Expanding mental health literacy also involves the development of culturally validated terminology so community members can appropriately discuss mental health and illness. The Samoa Nurses Association explained the importance of using Samoan cultural philosophy to create an essential understanding of mental health in a Samoan context; approaching mental health as “*soifua maloloina o le mafaufau*”, or a holistic state of well-being, can help Samoan families recognize the ways an adolescence’s wellness can be affected by a myriad of social factors [32].

Similarly, many KIs in our study believed that expanding mental health literacy in American Samoa would require the improvement of the current mental health infrastructure as well as the development of culturally validated terminology to discuss topics related to mental health. Since confidentiality was a frequently cited concern among KIs, it may be beneficial to increase trainings among mental health professionals while informing the public which available mental health services and resources in American Samoa are confidential; however, this could be challenging to address in a small setting where many individuals know one another [33].

Many participants also described several programs led by community leaders, youth, and school faculty that are aimed at increasing mental health awareness on the island. Skill-training and evidenced-based suicide prevention programs that were reported by KIs to be used in schools include *SOS*: *Signs of Suicide* and *Youth Mental Health First Aid* [16, 34–36]. Additionally, several nonprofit organizations and events like Pacific Roots Open Mic (PROM) and Empowering Pacific Islander Communities (EPIC) support adolescents through community outreach efforts and activities that promote self-confidence among youth [37, 38]. Similar school interventions and programs are being implemented in Samoa by NGO Fa’ataua Le Ola (“Make life important”), which also offers free 24/7 confidential phone counseling to Samoans [39]. Another mental health promotion initiative in Independent Samoa was the launch of a 12-month-long series of health-oriented art workshops that provided materials and resources for community members to exercise creative expression. The pilot study reported enhanced self-esteem, levels of confidence, and communication skills among participants, particularly among young women and child victims of domestic violence and sexual abuse, suggesting that a combined health and art initiative like art therapy may allow for higher public engagement with mental health promotion campaigns [40]. Future interventions that aim to increase mental health literacy among adolescents in American Samoa as well as parents and trusted adults may reduce mental health stigma and promote an environment where adolescents feel safe to seek social support for their mental health.

This qualitative study has several strengths and limitations. One notable limitation is that participants were recruited and interviewed during the COVID-19 pandemic and recently after a cluster of suicides had occurred on the island, two factors that may have resulted in a higher awareness and heightened emotion about mental health issues. At some levels of the socio-ecological model used here, adult KIs provided more input than adolescents (as evidenced in the quotes provided), although this may be expected given their relative positions in society and the likely lack of exposure of adolescents to some of the structural barriers to mental health care. The large sample size, especially given the size of the American Samoan population, did, though, include a diverse set of both adolescent and adult Samoan voices, and the community-partnered approach to this work allowed the involvement of many Samoan voices in the design and analysis phases. The semi-structured interviews and focus groups from a variety of participants offered detailed insight into the barriers to mental health care and the perceived stigmatization of mental health in American Samoa.

The current study shares a diverse range of community viewpoints on the experiences and stigmatization of mental health stigma among adolescents in American Samoa. These findings may inform local community leaders, the Department of Health, the Department of Education, and community-based organizations on the need to address and reduce mental health stigma when designing mental health interventions and services for adolescents.

### Do you need mental health support?

If you are struggling with your mental health, please call the +988 Suicide and Mental Health Helpline to connect with a mental health counselor in American Samoa and across the US.
